# Polyclonal non multiresistant methicillin resistant *Staphylococcus aureus* isolates from clinical cases of infection occurring in Palermo, Italy, during a one-year surveillance period

**DOI:** 10.1186/1476-0711-11-17

**Published:** 2012-06-19

**Authors:** Caterina Mammina, Cinzia Calà, Celestino Bonura, Paola Di Carlo, Aurora Aleo, Teresa Fasciana, Anna Giammanco

**Affiliations:** 1 Department of Sciences for Health Promotion “G. D’Alessandro”, University of Palermo, Palermo, Italy

## Abstract

**Background:**

The evolving epidemiology of methicillin resistant *Staphylococcus aureus* (MRSA) is characterized by the emergence of infections caused by non multiresistant MRSA carrying staphylococcal chromosomal cassette (SCC)*mec* IV or V in the healthcare settings. A molecular epidemiological analysis of non multiresistant MRSA isolates from four acute general hospitals was performed in Palermo, Italy, during a one year period.

**Methods:**

For the purpose of the study, MRSA isolates were defined as non multiresistant when they were susceptible to at least three classes of non β-lactam antibiotics. Seventy-five isolates were submitted to antimicrobial susceptibility testing, multilocus sequence typing (MLST) and polymerase chain reaction (PCR) for SCC*mec*, accessory gene regulator (*agr*) groups, arginine catabolic mobile element (ACME) and Panton Valentine leukocidin (PVL) toxin genes. For epidemiological typing, Multiple-Locus Variable-Number Tandem Repeat Fingerprinting **(**MLVF) was performed on all isolates and pulsed field gel electrophoresis (PFGE) on ST8 isolates.

**Results:**

Non multiresistant MRSA isolates were isolated from all hospitals. Resistances to ciprofloxacin, macrolides and tetracycline were the most prevalent. MLST attributed 46 isolates with ST22, 13 with ST8, eight with ST1, three with ST50 and three with ST398. SCC*mec* type IV was found in all isolates. PVL was detected in one ST22 isolate. All isolates tested negative for the ACME element. MLVF identified 31 different patterns, some subtype clusters ranging in size between two and 22 isolates. The closely related PFGE patterns of the ST8 isolates differed from USA300.

**Conclusions:**

A polyclonal circulation of non multiresistant MRSA along with blurring of boundaries between healthcare associated (HA)-MRSA and community associated (CA)-MRSA appear to be occurring in our epidemiological setting. A better understanding of spread of MRSA with the support of molecular typing can provide invaluable information in the epidemiological, microbiological and clinical fields.

## Background

Methicillin resistant *Staphylococcus aureus* (MRSA) is a leading cause of disease, such as skin and soft tissue infection (SSTI), pneumonia, bloodstream infection (BSI), osteomyelitis and endocarditis, as well as toxin-mediated syndromes [1]. MRSA is unquestionably one of the most important nosocomial pathogens worldwide, but recently it is increasingly identified as the etiological agent of infections acquired in community [1,2]. Although evidence has accumulated suggesting that some strains of community associated (CA)-MRSA might have spilled from healthcare setting in the community, molecular epidemiological studies indicate that CA-MRSA and healthcare associated (HA)-MRSA may have distinctive phenotypic and genetic features [1,2]. Traditionally, CA-MRSA are attributed with characteristics, such as smaller staphylococcal chromosomal cassette (SCC)*mec* cassettes – types IV and V – and a more restricted resistance pattern to antibiotics other than β-lactams than HA-MRSA [3]. However, recently, a bidirectional crossing of borders between HA- and CA-associated infections is occurring, with HA-MRSA clones being responsible for infections in community and, *vice versa*, CA-MRSA causing infections in hospitalized patients [4]. The changing epidemiology of MRSA is also been characterized by the emergence of infections caused by non multiresistant MRSA carrying SCC*mec* IV or V in the healthcare settings [3,5].

This study had the aim to perform a molecular epidemiological analysis of all non multiresistant MRSA isolates carrying SCC*mec*IV from out- and inpatients admitted to all wards of four acute general hospitals in Palermo, Italy, during the period February 1, 2009 - January 31, 2010.

## Methods

### Bacterial isolates

This study was conducted in the period February 1, 2009 – January 31, 2010 with the collaboration of the clinical microbiology laboratories of three acute general hospitals and one teaching hospital in Palermo, Italy.

Overall, isolates from 179 inpatients and ambulatory outpatients were collected and sent to the coordinating laboratory at the Department of Sciences for Health Promotion “G. D’Alessandro”, University of Palermo, Italy. Only one isolate per patient was included in the study. In particular, all the unique isolates were analyzed. In the event of multiple consecutive isolations from the same patient, only the first isolate since the admission was included. Isolates from infection control screening specimens were excluded.

Information about ward and type of biological sample was obtained, whereas clinical and epidemiological information regarding patient’s risk factors and previous or ongoing antimicrobial therapy was generally unavailable.

At the coordinating laboratory, all MRSA isolates were sub-cultured for purity on mannitol-salt agar. Their identification was confirmed by biochemical tests, coagulase production and the cefoxitin disk diffusion test, according with the interpretive criteria of the Clinical and Laboratory Standards Institute (CLSI) [6]. Submitted isolates exhibiting equivocal results were confirmed by detection of *mecA* gene by use of polymerase chain reaction (PCR). The isolates were stored in glycerol at −70°C.

For the purpose of the study, MRSA isolates were defined as non multiresistant when they were susceptible to at least three classes of non β-lactam antibiotics (aminoglycosides, fluoroquinolones, macrolides, rifampicin, sulfamethoxazole-trimethoprim, tetracyclines).

### Multilocus sequence typing (MLST)

MLST was performed, as previously described, for a selected group of representative strains of each MLVF pattern [7]. The MLST allelic profiles and sequence types were assigned by submission to the *S. aureus* MLST database (http://www.mlst.net).

### Polymerase chain reaction (PCR)

Determination of SCC*mec* types was performed by multiplex PCR, according with Zhang et al. [8] and Milheiriço et al. [9]. Accessory gene regulator (*agr*) specificity grouping was carried out for all MRSA isolates [10]. MRSA isolates were further defined by the presence of arginine catabolic mobile element (ACME) and the Panton-Valentine leukocidin (PVL) toxin genes by PCR for *arcA* and *lukS-PV*, respectively [11]. An USA300 reference strain was kindly provided by Prof. S. Stefani, Molecular Microbiology and Antibiotic Resistance Lab, Department of Microbiology, University of Catania, Catania, Italy, and included in the analysis.

### Antimicrobial susceptibility testing

Participating laboratories provided susceptibility patterns obtained by their routine methods. Minimum inhibitory concentrations of vancomycin, teicoplanin, tigecycline, daptomycin and linezolid were re-assessed by Etest (AB Biodisk, Solna, Sweden). All assays were performed and interpreted in accordance with Clinical and Laboratory Standards Institute (CLSI) guidelines [6].

### Multiple-locus variable-number tandem repeat fingerprinting (MLVF)

The set of PCR primers described by Sabat et al. [12] was used to simultaneously amplify the hypervariable variable number tandem repeat (VNTR) regions of the *spa*, *sspA*, *clfA*, *clfB* and *sdr* genes with the modifications proposed by Karinsky et al. [13]. Amplification and gel electrophoresis conditions were as previously described [12]. The MLVF patterns were analyzed by using BioNumerics v 5.01 software (Applied Maths, Sint-Martens Latem, Belgium). A dendrogram was produced by using Dice coefficients and unweighted pair-group method using geometric averages (UPGMA), with 0.5% optimization and 1.25% band position tolerance [13]. The results obtained were confirmed by visual inspection. Any two MLVF patterns differing by at least one band were considered distinct types and attributed with sequential numerical codes.

### Pulsed field gel electrophoresis (PFGE)

ST8 MRSA isolates representative of the different MLVF patterns and the USA300 reference strain were genotyped by PFGE after *Sma*I digestion of chromosomal DNA, prepared using the protocol described by Cookson et al. [14] with slight modifications. The banding patterns were visually assessed and interpreted according with previously published criteria [15].

## Results

In the period under study, 75 (41.9%) non multiresistant MRSA isolates were identified among a total of 179 isolates from the four hospitals participating to the study. Non multiresistant MRSA isolates were isolated from all hospitals: 28 each from the hospitals A and D, 11 from the hospital C and eight from the hospital B, respectively. Thirty-six were isolated from inpatients admitted to medical wards, 21 from intensive care unit (ICU) patients, nine from surgical patients, and four only from outpatients. As summarized in Table 1, the most common sites of infection were respiratory tract (28 cases), skin and soft tissues (23 cases), and bloodstream (10 cases). In five cases, no information about the clinical sample was available.

**Table 1:** Distribution of the sites of infection among the different sequence types (STs) of the MRSA isolates under study

**Site of infection**	**No. of isolates**				
	ST1	ST8^a^	ST22	ST50	ST398
Respiratory tract	5	8	13	1	1
Skin and soft tissues	2	3	16	-	2
Bloodstream	-	3	6	2	-
Eye	-	-	3	-	-
Surgical site	1	-	2	-	-
Urinary tract	-	1	1	-	-
Unknown	-	-	5	-	-

^a^ including ST1517.

MLST showed that 46 isolates belonged to ST22, 13 to ST8, eight to ST1, three to ST50 and three to the livestock associated MRSA (LA-MRSA) ST398 clone. Two further isolates proved to belong to ST1517, a single locus variant (SLV) of ST8. CA-MRSA belonging to the major European clone ST80 were not identified.

As detailed in Table 1, SCC*mec* type IV was found in all MRSA isolates. The most common profile was SCC*mec* subtype IVa (51 isolates, 68.0%), followed by SCC*mec* IVb (13 isolates) and SCC*mec* IVc (11 isolates). Thirty-three out of 46 isolates, belonging to ST22-IV (EMRSA-15) carried SCC*mec* IVa, whilst the remaining 13 SCC*mec* IVb. Furthermore, SCC*mec* IVc was identified in most ST8-IV isolates (11 out of 15 isolates) and SCC*mec* IVa in the four remaining isolates, including those attributed with ST1517. All ST1, ST50 and ST398 isolates carried the subtype SCC*mec* IVa.

ST22, ST8 and ST398 isolates belonged to *agr* group I, whereas ST1 and ST50 isolates were attributed, respectively, with *agr* groups III and IV. PVL was detected in only one isolate belonging to ST22 from a SST infection. All isolates tested negative by PCR for the *arcA* locus of the ACME element.

Table 2 illustrates the resistance profiles of the MRSA strains under study. In particular, 36 isolates (48.0%) were resistant to ciprofloxacin, 35 (46.7%) to chlarytromycin, 32 (42.7%) to erythromycin, and 23 (30.7%) to tetracycline. Resistance to gentamicin (five isolates), sulfamethoxazole-trimethoprim (three isolates) and rifampicin (three isolates) proved to be less frequent. All strains tested susceptible to vancomycin, teicoplanin, linezolid, daptomycin and tigecycline. Twenty-three out of 46 of the ST22 isolates and 13 out of 15 ST8/ST1517 isolates proved to be resistant to ciprofloxacin, in comparison with none of ST1 and ST50 isolates. One ST398 isolate was also ciprofloxacin resistant. Resistance to macrolides was also frequent and associated with some clustered isolates, such as ST22-MRSA-IVb MLVF 023 and 073, and those belonging to ST1.

**Table 2:** Distribution of the MRSA isolates on the basis of the molecular epidemiological characterization and pattern of resistance to non β-lactam antibiotics

**No. of isolates**	**ST**^**a**^	**SCC*mec*type**	**No. of isolates**	**Resistance profile**^**b**^	**MLVF**^**c**^**type**
46	22	IVa	10	TET	004
			2	CLR TET	004
			2	CIP	004
			1	CIP TET	004
			1	GM TET	004
			6	susceptible	004
			1	CIP SXT	009
			1	CIP CLR ERY	017
			1	CIP CLR ERY	019
			1	susceptible	024
			1	susceptible	043
			1	CIP CLR ERY	058
			1	CIP	059
			1	SXT TET	060
			1	CIP ERY	061
			1	CIP	068
			1	TET	070
		IVb	7	CIP CLR ERY	023
			5	CIP CLR ERY	073
			1	CIP CLR ERY SXT	073
13	8	IVc	2	CIP CLR ERY	006
			2	CIP	044
			2	CIP CLR ERY	044
			1	CIP ERY	012
			1	CIP GM RIF	018
			1	CIP ERY	035
			1	CIP CLR ERY GM	045
			1	CIP	064
		IVa	1	susceptible	003
			1	CIP CLR ERY	045
8	1	IVa	3	CLR ERY TET	065
			2	CLR ERY TET	033
			1	CLR ERY	010
			1	CLR ERY	040
			1	CLR TET	049
3	50	IVa	2	susceptible	030
			1	susceptible	002
3	398	IVa	1	CLR GM RIF TET	029
			1	CIP TET	029
			1	GM RIF TET	029
2	1517	IVa	2	CLR	057

^a^ ST, sequence type.

^b^ other than β-lactams.

CLR, clarithromycin; CIP, ciprofloxacin; ERY, erythromycin; GM, gentamicin; RIF, rifampicin; SXT, sulfamethoxazole- trimethoprim; TET, tetracycline.

^c^ MLVF, Multiple-Locus Variable-Number Tandem Repeat Fingerprinting.

**Figure 1:**
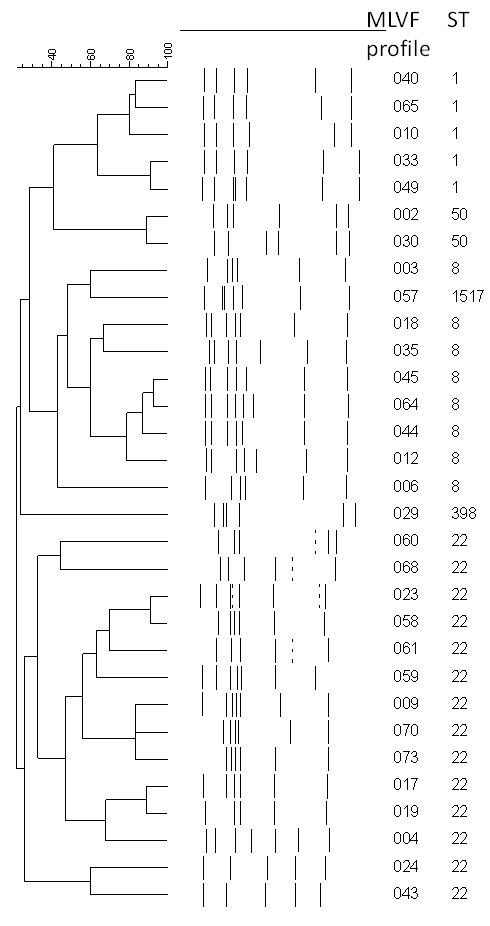
** Dendrogram showing similarity between the 31 different Multiple-Locus Variable-Number Tandem Repeat Fingerprinting (MLVF) patterns.** Sequence type (ST) is also indicated.

To obtain a more discriminative picture of the MRSA strains in the four hospitals under study and highlight possible subtype clusters of epidemiological interest, all MRSA were submitted to MLVF. This fingerprinting technique allowed for the identification of 31 different banding patterns among the 75 isolates under study (Table 2 and Figure 1). Some subtype clusters were also recognized ranging in size between two and 22 isolates. In particular, the largest subtype cluster, including 22 isolates, was characterized by the MLVF pattern 004 and was found within the ST22-IVa isolates. It comprised isolates with six different antibacterial susceptibility patterns from all the participating hospitals. Moreover, two smaller clusters, grouping seven and six isolates, respectively, were identified among the ST22-IVb isolates: the first one, characterized by the MLVF pattern 023 contained isolates from three out of the four hospitals under investigation, whereas the second one with the MLVF pattern 073 consisted of isolates that, except for one of them, had been isolated from patients attending different wards of the same hospital.

No significant subtype clusters were identified among the ST8/ST1517-IV isolates. Three couples of strains with identical MLVF patterns and drug resistance profiles were indeed recognized, but four of them, showing the profile 044, came from different hospitals. The two isolates ST1517 shared the same MLVF profile 057, but they were isolated within a few days of each other from an inpatient and an outpatient, respectively, without any meaningful epidemiological correlation.

Of special interest, an indistinguishable MLVF pattern was shared by three ST398-MRSA-IVa isolates. They were identified several months apart from each other (February 2009, July 2009 and January 2010) at the same hospital from a patient with ventilator-associated pneumonia and two further patients with serious SST infections.

The ST8 MRSA isolates shared indistinguishable or closely related PFGE banding patterns, but distinctly different from that of USA300 strain.

## Discussion

MRSA is one of the most prevalent pathogens isolated from hospitalized patients and is increasingly identified in outpatient settings. Along with the spread of some multiresistant clones in the healthcare settings, emergence of CA-MRSA is cause of major concern for both clinicians and public health specialists [3]. Blurring of boundaries between HA-MRSA and CA-MRSA is a further challenge predominantly arising from changes in the hospitalized patients management. This involves increased bed turnover and patient throughput and the more and more complex patterns of transfer of patients within the healthcare network including teaching and non teaching hospitals, long term care facilities, nursing homes, home care and other alternative healthcare settings [4]. Moreover, the selective pressure operated by antibiotic use is likely contributing also to the acquisition and/or loss of virulence genes carried on mobile genetic elements that can easily spread within and between lineages [16].

SCC*mec* cassette IV and a non multiresistant pattern of drug susceptibility generally characterize some well known clones of HA-MRSA, such as ST22-MRSA-IV, a pandemic CC22-MRSA strain known as (UK-)EMRSA-15, and the CA-MRSA strains. It has been, on the other hand, debated about the ecological advantage in terms of bacterial fitness provided to these strains by a “light” SCC*mec* cassette, that in part could explain their worldwide successful spread [17,18].

In our one-year surveillance period, ST22-MRSA-IV proved to be the most prevalent non multiresistant clone in Palermo, Italy. It was identified, indeed, in all the hospitals under investigations, in many different wards and clinical samples. It proved also to include as much as 20 different strains based upon their MLVF banding and drug susceptibility patterns. It is noteworthy also that during the surveillance period an outbreak of colonization/infection by EMRSA-15 with the MLVF pattern 004 occurred in the NICU of one out of the four hospitals [19]. EMRSA-15 emerged in the United Kingdom in 1991, spread widely, and in the following years accounted for more than 90% of MRSA isolates [20]. It is being detected in hospitals as well as in outpatients in several geographic areas, such as Europe, where it appears to be replacing previous established clones. Indeed, EMRSA-15 has been found in a number of hospitals in the Netherlands [21], in the Czech Republic [22], in Portugal [23], as well as in Malta [24]. EMRSA-15 has been also previously described as an emerging clone in some Italian regions [25]. Ability of strains belonging to this clone to survive long time on inanimate surfaces is likely providing a better opportunity to spread within healthcare settings and toward the community than other HA-MRSA clones [26]. A role has been also suggested for the diffusely increasing use of fluoroquinolones that is likely exerting a selective pressure favoring the emergence of MRSA strains that are usually resistant to these drugs, such as EMRSA-15 [27]. However, according with previous reports [28], ciprofloxacin susceptibility was not a so infrequent event, involving 50% of the ST22 strains. As a consequence, when screening for MRSA by using ciprofloxacin-containing selective media, caution is to be adopted. Consistently with reports from other countries [29,30], but unlikely from previous data from Italy [25], in our experience the only MRSA isolate that tested positive to PVL proved to belong to ST22.

The second most represented clone in our surveillance study was ST8-MRSA-IV. Clonal type ST8 associated with different SCC*mec* types has been previously described mainly in association with CA-MRSA strains. However, all of our strains were *pvl*/*arcA* negative and had PFGE pattern not identical to that of USA300. Thus, on the basis of their genetic characteristics, the ST8 isolates were presumed to belong to the HA-MRSA epidemic clone (UK) EMRSA-2/6 [31]. Resistance to ciprofloxacin is also consistent with previous reports about this clone [32]. ST8-MRSA-IV isolates with similar characteristics have been recently reported in a collection of isolates from 19 Italian hospitals in the 2000’s [33], as well as in other countries of the European area, such as Netherlands, [34] and of the Mediterranean basin, such as Israel [31,34].

ST1-MRSA-IV is a community associated clone. It has been recently documented as the most frequently identified PVL positive MRSA clone in skin and soft tissue infections in some communities of northern Canada [35]. However, PVL negative strains also have been reported to cause serious infections in high risk groups such as intravenous drug users [36]. In our experience, ST1 isolates appeared to be associated not only to SSTIs, but mainly to respiratory tract infections. They appeared also rather homogeneous by drug susceptibility pattern and MLVF banding profiles. At our best knowledge this is the first report of healthcare associated human infections by ST1-MRSA-IVa in Italy. Interestingly, ST1 strains but with distinctive properties, such as several virulence and resistance genes towards major classes of antimicrobials, SCC*mec* type V and peculiar PFGE patterns, have been recently reported to circulate in holdings of breeding pigs in Italy [37].

Similar considerations can be done about the first detection in Italy of three isolates of ST50-MRSA-IVa, a further CA-MRSA clone, that has been identified in hospitalized patients during the period under study. Although for two out of them with a MLVF pattern 030, that had been isolated in two patients in the same ward one month distant from each other, a intrahospital cross-transmission event could be reasonably hypothesized, presence of this additional CA-MRSA clone in the hospital setting is cause of further concern.

A special attention deserves the identification within the MRSA isolates recovered during the one-year period of surveillance of three ST398-MRSA-IVa strains. It has become apparent since some years that livestock constitutes a reservoir for this MRSA clone and can be a potential source of transmission to humans [1-3]. In particular, the frequent colonization of livestock with MRSA in many European countries is a cause for great concern, even if knowledge and understanding mechanisms of emergence and spread of these organisms and their public health implications are still preliminary. According with some authors, attributing mechanically human infections with *S. aureus* ST398 to livestock reservoirs at this time still needs prudence and the hypothesis that ST398 variants might persist in humans without livestock contact cannot be discharged [38]. Possible role as ST398 MRSA vehicle of meat or other food products of animal origin is under scrutiny [39]. Prevalence of ST398 MRSA within the clinical isolates under study was 1.7%, a similar proportion to that recently reported for some European countries, such as Austria and Denmark [40]. In our experience, ST398-MRSA-IVa was isolated from three different patients, of whom two outpatients, without any epidemiological relationship between each other. Of further concern, two of them reported no history of livestock or pet exposure.

MLVF was thoroughly adopted for epidemiological typing based upon previous reports confirming stability and adequacy of the method to a broad use in detection of outbreaks and potential sources of transmission [12,41] and its high discriminatory power [41,42]. Additionally, the MLVF method is a cost-effective and speedy tool suitable for application in routine microbiology laboratories already applying PCR-based assays. Our data confirm the ability of MLVF to subtype the more prevalent MRSA lineages. In particular it was able to distinguish between subtypes of ST22 an ST8 and unambiguously identify ST398 strains.

To conclude, some considerations arise from the results of our study:

circulation of non multiresistant MRSA in our epidemiological setting proved to be polyclonal

epidemiology of MRSA is evolving in our geographic area toward the blurring of boundaries between HA-MRSA and CA-MRSA, that has been largely documented in other developed countries. Detection of “true” CA-MRSA clones, such as ST1 or ST50 in inpatients is a clear evidence of this ongoing phenomenon deserving further strict monitoring

because of the high prevalence of EMRSA-15, detection of SCC*mec*IV is not a good predictor of CA-MRSA

PVL-negative CA-MRSA strains are circulating in hospitalized patients in western Sicily, a finding that has been previously described in other Italian regions. Virulence of these strains is reasonably expected to be lower, but understanding of clinical and epidemiological implications of this event is still very limited

recovery of ST398 from three human cases of infections, two of whom with a negative history of contact with livestock, adds a further element of concern.

## Conclusions

Understanding epidemiology of MRSA is indispensable to guide targeted initiatives to control spread of these organisms in the healthcare settings and the community. Surveillance of MRSA clones by using molecular typing can provide invaluable information in the epidemiological, microbiological and clinical fields. In this respect, local epidemiological data may support control efforts aimed at interrupting the spread within and between different healthcare settings and between these and the community. Concurrently, they may contribute to build and update surveillance databases at both national and international levels.

## Competing interests

The authors declare that they have no competing interests.

## Authors’ contributions

CC, CB, AA and TF performed molecular typing, assessment of genetic relationships among strains and interpretation of the results. CM, PDC and AG participated in the study design and analysis of the data. CM and AG drafted the manuscript. The components of the EPI-MRSA Working Group collected data, performed identification and antibiotic susceptibility testing, stored the isolates and provided information about them. All authors read and approved the final manuscript.

## Authors’information

EPI-MRSA Working Group: Daniela Maria Geraci^*^, Maria Antonietta Saporito^†^, Concetta Sodano^†^, Maria Stella Verde^†^, Rosa Lia Genco^‡^, Anna Maria D’Accardo^§^, Teresa Amato^#^, Enza Di Carlo^#^, Salvatore Distefano^#^, Rita Immordino^#^, Laura Pitarresi^#^, Roberta Virruso^#^

^*^Department of Sciences for Health Promotion “G. D’Alessandro”, University of Palermo, Italy

^†^Laboratory of Microbiology, ARNAS, General Hospital “Civico e Benfratelli”, Palermo, Italy

^‡^Laboratory of Microbiology, General Hospital “Buccheri-La Ferla”, Palermo, Italy

^§^Laboratory of Microbiology, General Hospital “V. Cervello”, Palermo, Italy

^#^Laboratory of Microbiology, Teaching Hospital “Azienda ospedaliero-Universitaria Policlinico “Paolo Giaccone”, Palermo, Italy
